# Supplementary material for: Defining a conformational ensemble that directs activation of PPARγ
Source: Nat Commun. 2018 May 4;9:1794. doi: 10.1038/s41467-018-04176-x (PMC5935666; doi:10.1038/s41467-018-04176-x)
Supplement: Supplementary file 1 — Supplementary Information [file 41467_2018_4176_MOESM1_ESM.pdf]

Supplementary Information for:

Defining a conformational ensemble that directs activation of PPARγ, Chrisman et al.

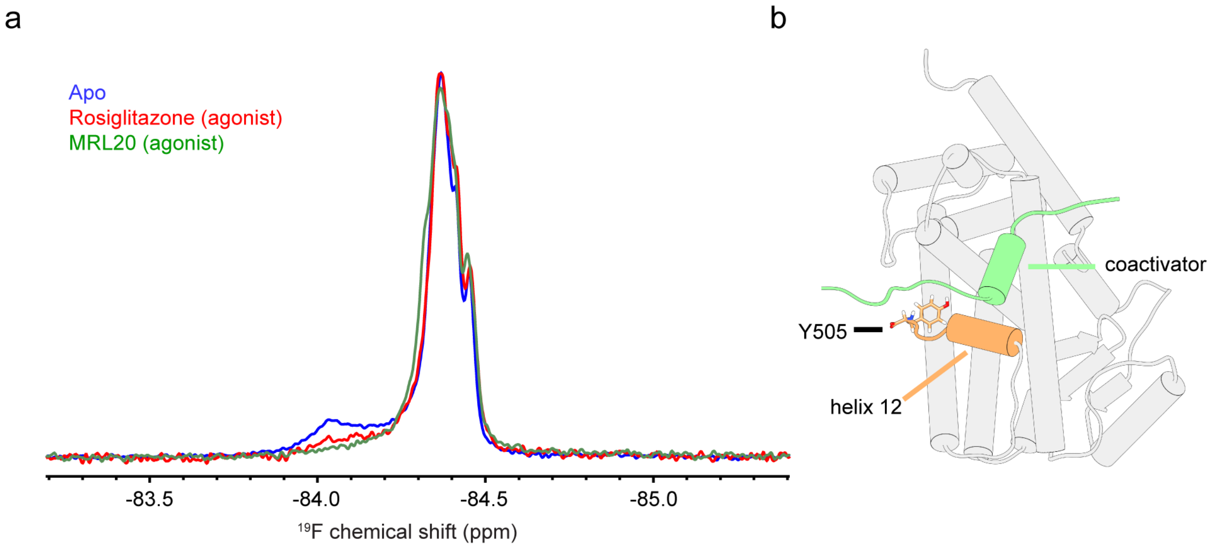

Supplementary Figure 1 | The fluorine NMR spectrum of Y505C C313S PPARγ LBD is largely ligand independent. a) Y505C C313S PPARγ LBD was labeled with BTFA and fluorine NMR was performed at ~376 MHz at room temperature either in apo form (blue spectrum) or in the presence of two agonists (rosiglitazone; red and MRL20; green). b) Y505 is located at the unstructured C-terminus of the PPARγ LBD, which likely contributes to the relative lack of change upon ligand binding. This protein was not delipidated.

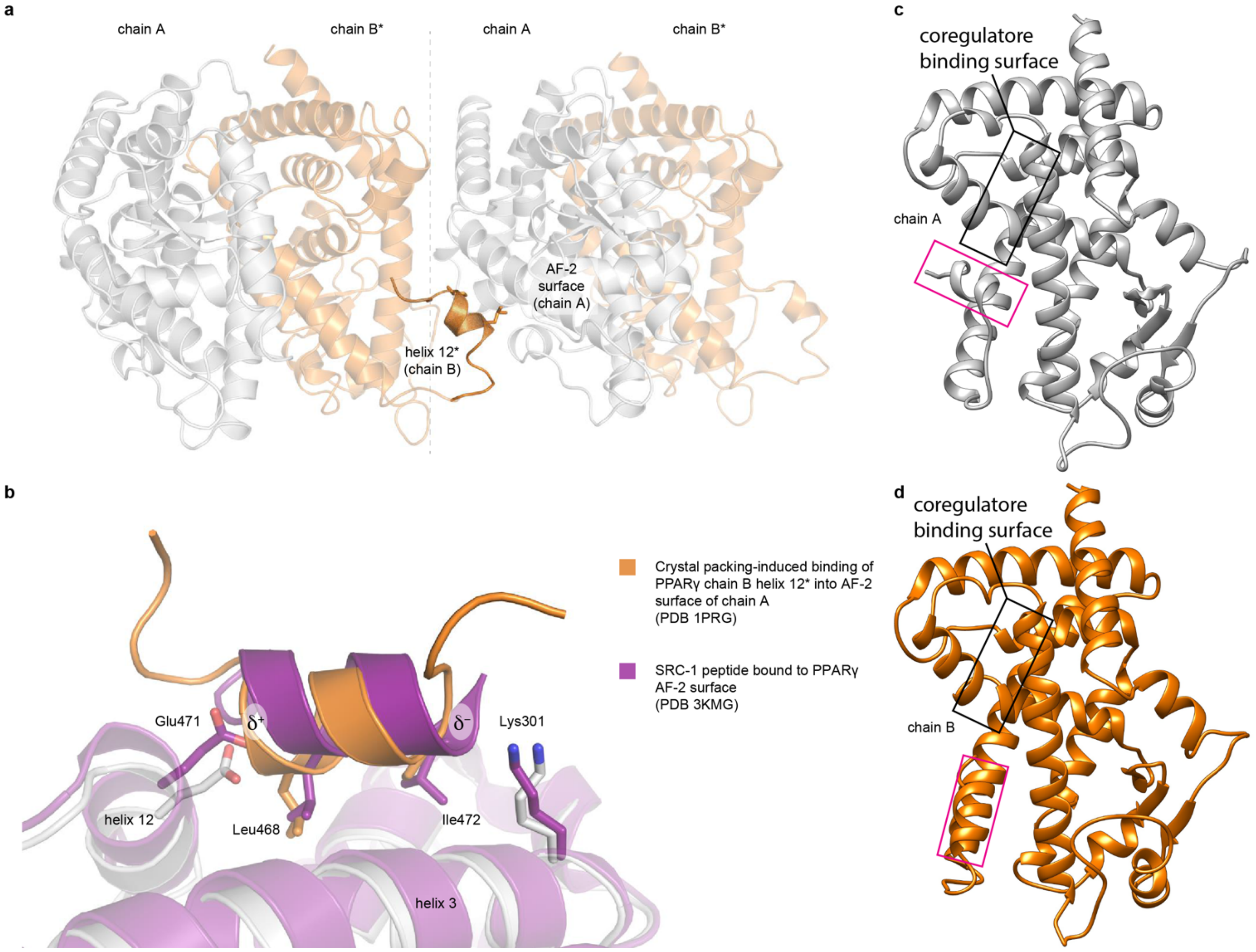

Supplementary Figure 2 | Influence of crystal packing on PPARγ LBD helix 12 conformation. The PPARγ LBD often crystalizes as a homodimer where helix 12 of the symmetry-related B chain (helix 12*) associates with the AF-2 surface of the A chain (**a**), adopting a conformation similar to a coactivator peptide bound to the AF-2 surface (**b**). This crystallization artifact distorts the conformation of the B chain helix 12* and influences the conformation of the A chain helix 12 through the formation of "charge clamp" hydrogen bonds with a positively charged (Lys301) of A chain helix 3 and negatively charged (Glu471) residues of A chain helix 12. This creates a dipolar coactivator mimicking protein-protein interaction (δ+/δ-) between monomeric subunits that is further stabilized by hydrophic interactions originating from Leu468 and Ile472 of B chain helix 12*, which biases the observed structure into an "active" (A chain) or "pseudo-inactive" (B chain) helix 12 conformation. (c) Chain A and (d) chain B forms of PPARγ (PDB code: 1PRG) with helix12 and the coregulator binding surface highlighted.

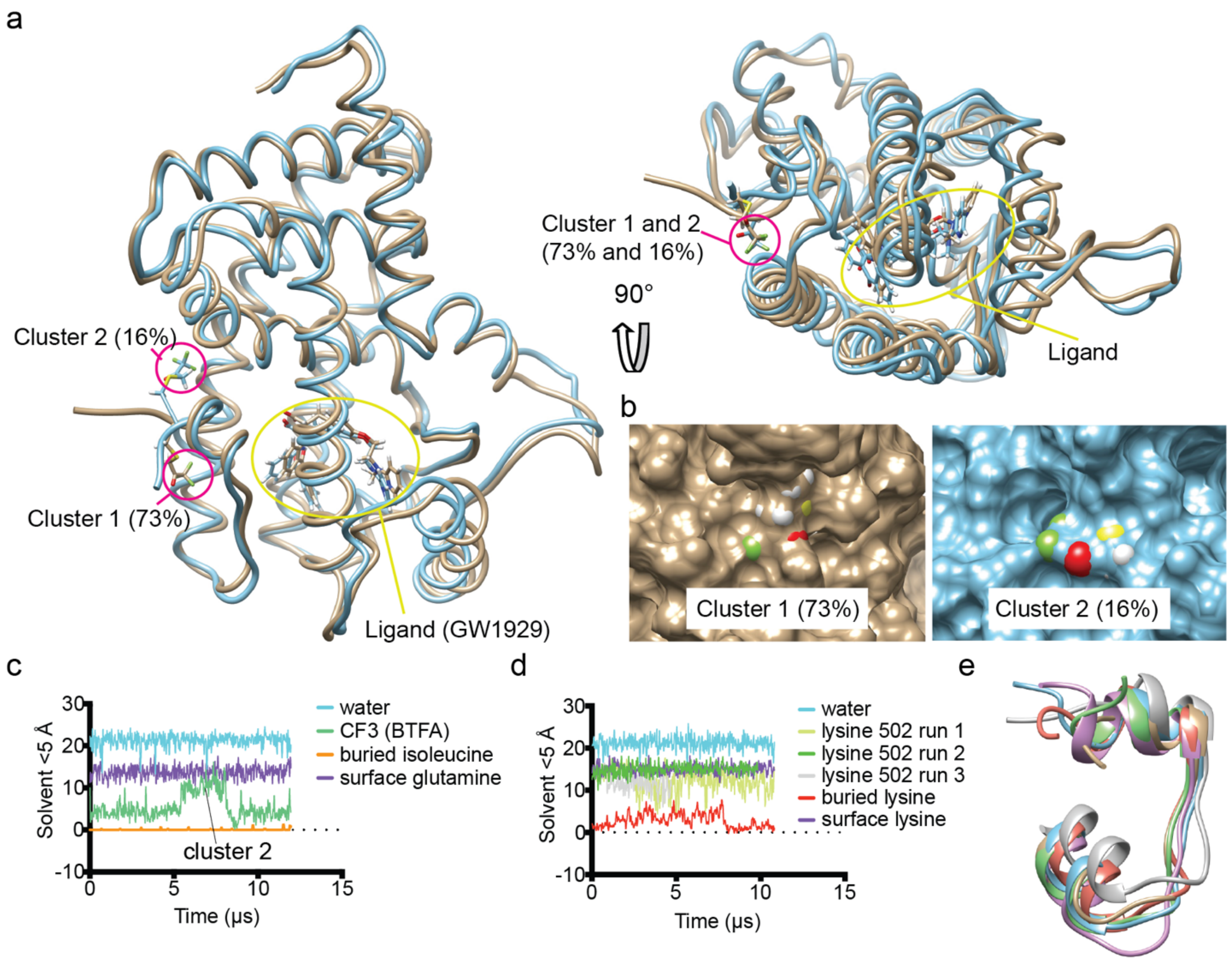

Supplementary Figure 3 | Molecular dynamics simulations indicate that the "agonist" helix 12 crystal conformation results in a solvent exposed side chain zeta nitrogen of residue K502 and terminal carbon of K502C-BTFA. (**a**) PPARγ^{K502C}-BTFA bound to GW1929 was simulated in explicit water and salts for ~12 μs and clustered into five clusters by label (cysteine-BTFA) position. Representative structures from the two most populated clusters (1 and 2 89% of total frames) are shown. The percent of the total simulation time spent in each cluster is highlighted. The trifluoromethyl of the BTFA label and the ligand are highlighted. Fluorine atoms are green. The remaining 11% of the frames are similar to those in cluster 1 and 2 except for a cluster of 3% of the frames where the BTFA is near the ligand and has very low solvent exposure. **(b)** The CF_3 group of K502C-BTFA is on the surface of the protein in these two clusters (**c**) Solvent exposure analysis of the PPARγ^{K502C}-BTFA simulation. The number of solvent molecules (water and ions) within 5 angstroms of the trifluoromethyl carbon of BTFA, and terminal carbons on the indicated amino acids and a randomly chosen water molecule are shown. (**d**) Three independent simulations were performed of GW1929 bound to PPARγ LBD totaling ~25 μs. The number of solvent molecules (water and ions) within 5 angstroms of the zeta Nitrogen of K502, other control lysines, including one pointing into the ligand binding pocket (buried Lysine), and a randomly chosen water molecule are also shown. (**e**) Representative structures from clustering of the PPARγ^{K502C}-BTFA simulation according to helix 12 RMSD into five clusters along with a crystal structure of an "active" helix 12 conformation (1PRG chain A; grey).

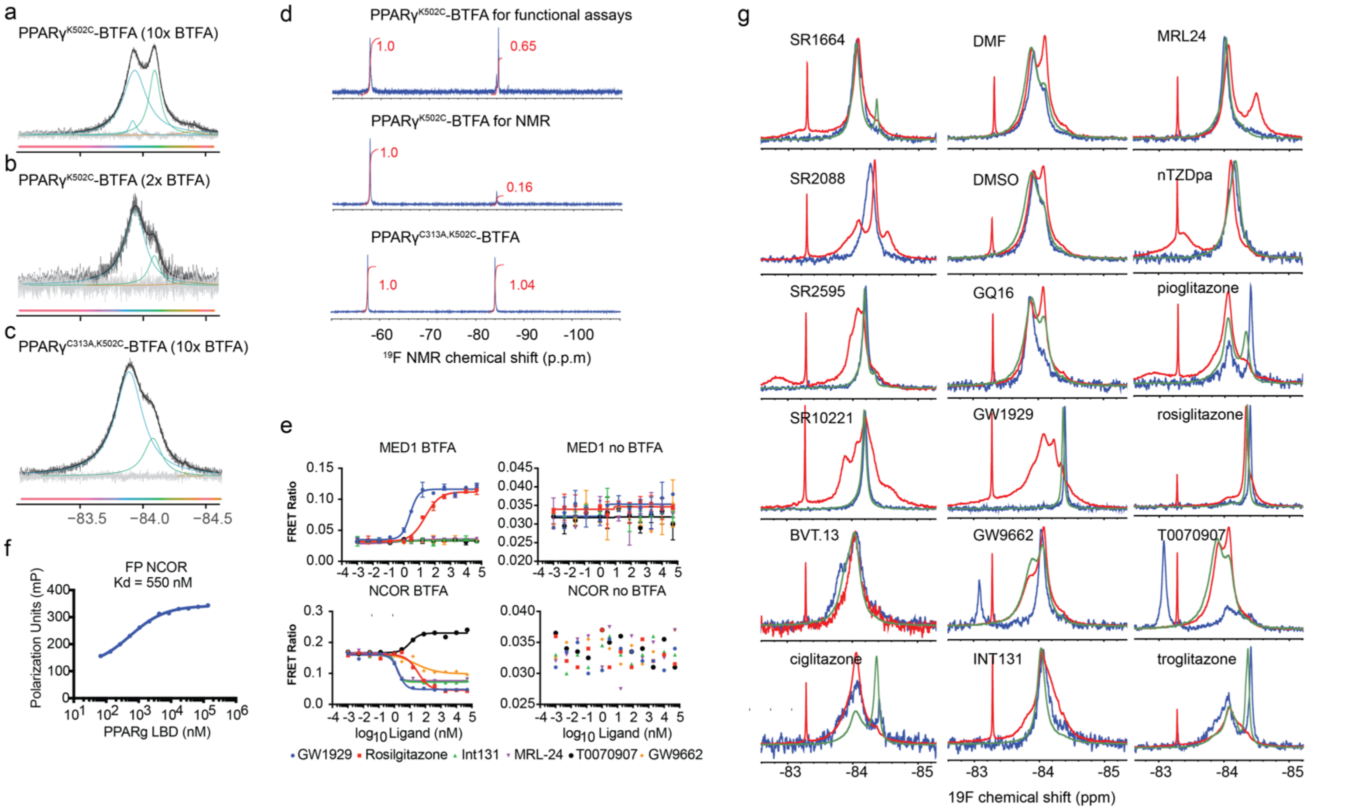

Supplementary Figure 4 | ^{19}F NMR indicates that K502C is preferentially labeled over C313 by BTFA and PPARγ^{K502C} without BTFA label is not active in TR-FRET. PPARγ^{K502C} which contains a native cysteine (C313), and an introduced cysteine (K502C) was incubated with either (a) 10x or (b) 2x molar ratio of BTFA. (c) PPARγ^{C313A,K502C} which contains a single cysteine (K502C) was incubated with 10x BTFA. The PPARγ^{C313A,K502C}-BTFA spectrum is very similar to the 2x BTFA treated PPARγ^{K502C} in panel b indicating that K502C is labeled preferentially. The spectra shown in panel b and c are shown in other figures in this manuscript (d) PPARγ^{K502C} was prepared for use in functional assays (TR-FRET and FP) or NMR and loaded with either 1.1 to 1.25 molar equivalents of MRL24 (which contains a CF_3 group; left peak). The signal from MRL24 (left peak) and BTFA (right peak) were integrated (red numbers in figure). The integral of the left peak indicates the amount of protein, while the right peak indicates the fraction labeled. (e) Ligand dependent recruitment of NCOR or MED1 peptide to PPARγ^{K502C} (no BTFA) or PPARγ^{K502C}-BTFA was measured using TR-FRET for select ligands. The failure of non-BTFA labeled PPARγ^{K502C} to produce a TR-FRET signal was also seen in two other separate TR-FRET experiments using GW1929. Mean of two technical replicates and standard deviation are shown. (f) The non-BTFA labeled PPARγ^{K502C} used in panel e has the expected affinity for NCOR as measured by fluorescence polarization (also see **Supplementary Figure 7b**) (g) Comparison of PPARγ^{C313A,K502C}-BTFA (green; only labeled on C502) with PPARγ^{K502C}-BTFA2x that was loaded *first* with indicated ligands and then exposed to a 2x molar ratio of BTFA (blue; presumably labeled only on C502) and PPARγ^{K502C}-BTFA10x that was exposed to a 10x molar ratio and then loaded with the indicated ligands (red; presumably labeled on both C502 and C313). PPARγ^{K502C}-BTFA2x is more similar to PPARγ^{C313A,K502C}-BTFA than PPARγ^{K502C}-BTFA10x in all cases except for the ligands which covalently attach to C313. (Dimethylformamide; DMF and Dimethyl sulfoxide; DMSO). The narrow peak at ~-83.3 ppm is free BTFA. All spectra displayed in panel g except for the red spectra are displayed in other figures in this manuscript.

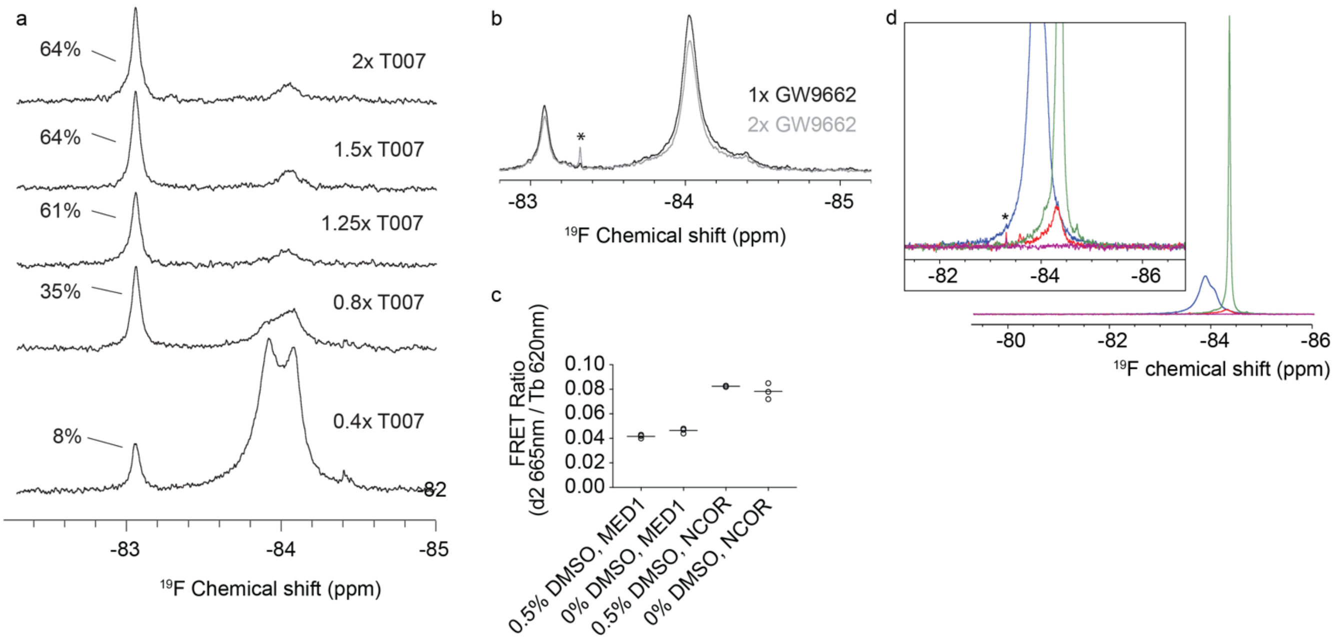

Supplementary Figure 5 | Covalent ligands do not bind to C502, DMSO has a minimal effect on TR-FRET, and signal from any BTFA labeled contaminating protein is undetectable. (a-b) The indicated molar ratios of a) T0070907 or b) GW9662 were added to PPARγ^{K502C} followed by labeling with 10x BTFA. The percent of the total signal area found in the left peak is indicated in panel a. (c) TR-FRET demonstrates that ligand vehicle (DMSO) has negligible impact on coregulator recruitment to apo PPARγ LBD. Three technical replicates are shown along with the mean. (d) PPARγ^{C313A} LBD (no cysteines) without (purple) or with (red) 6x histidine tag was treated with 10x molar ratio of BTFA and measured with fluorine NMR using the same processing and acquisition parameters as PPARγ^{C313A,K502C}-BTFA not bound to ligand (blue) or bound to GW1929 (green). Cleavage of the 6x histidine tag increases the purity of the protein because after cleavage the prep is re-run over the nickel column and contaminating proteins stick to the column while PPAR flows through. The difference in purity that histidine cleavage makes is large, with virtually no contaminating protein detected after cleavage. All spectra shown in this work except supplementary figure 2 used proteins with the histidine tag cleaved off, thus, NMR signal from impurities would not be expected to be observed, however any signal from protein impurities would be expected to be broad and centered at ~-84.35ppm as shown here. A small broad upfield shifted peak around ~-84.35 ppm appears inconsistently in deconvolutions of some spectra. As demonstrated by this control, it is possible, but not likely, that this signal near -84.35 ppm originates from a small amount of contaminating protein that is labeled with BTFA. Inset displays zoomed view of main figure. The * denotes signal from free BTFA. The blue and green spectra in panel d and the spectra in panel b are displayed in other figures in this manuscript.

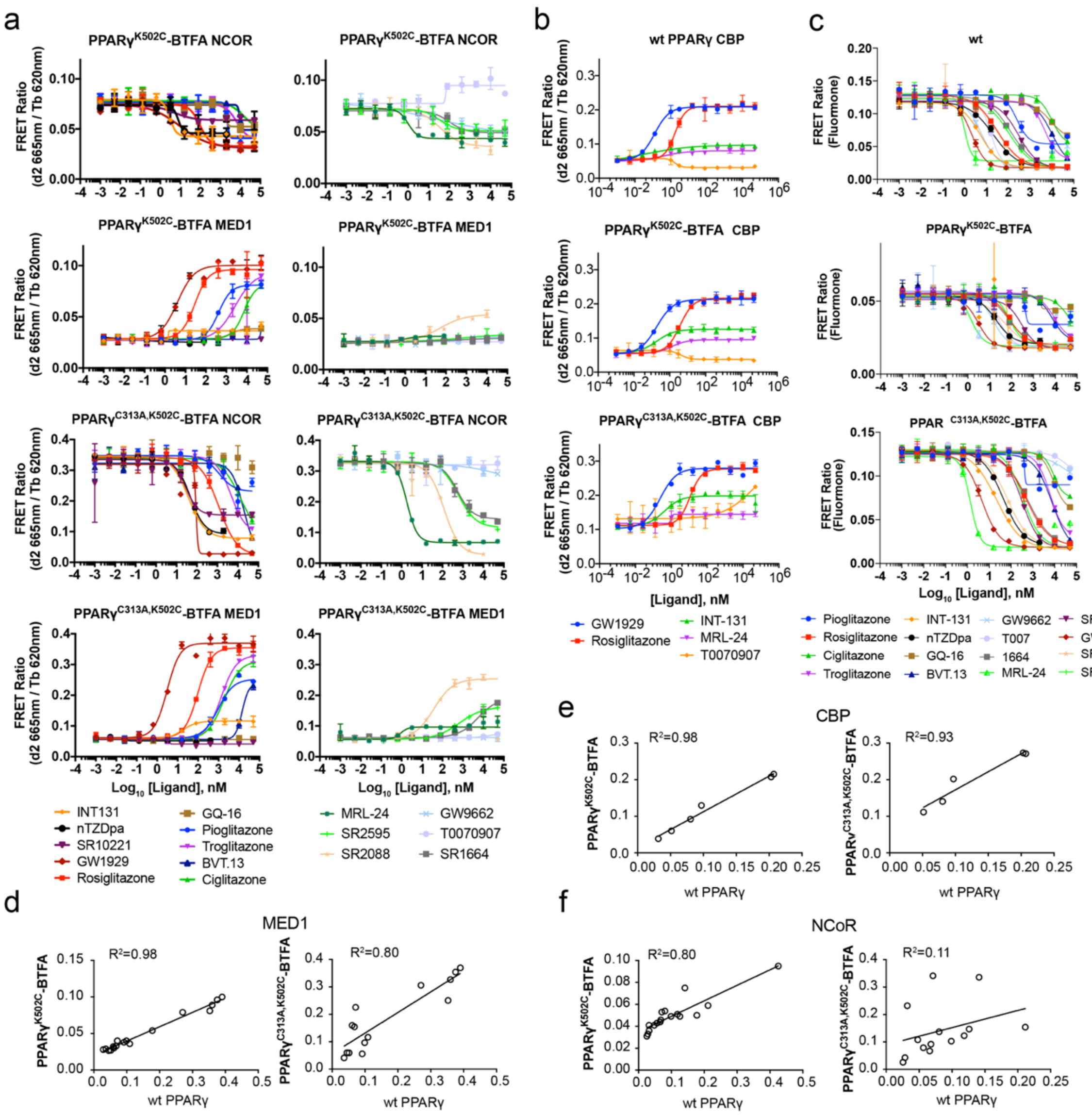

Supplementary Figure 6 | TR-FRET indicates that labeled proteins are functional, that PPARγ^{K502C}-BTFA is most functionally similar to PPARγ. (a) Titration of PPARγ^{C313A,K502C}-BTFA and PPARγ^{K502C}-BTFA with a diverse set of ligands in the TR-FRET assay using NCoR or MED1 biotinylated peptides and His-tagged PPARγ LBD (EC_{50} values are shown in **Supplementary Table 3**). (b) Titration of PPARγ, PPARγ^{C313A,K502C}-BTFA and PPARγ^{K502C}-BTFA with a subset of ligands in the TR-FRET assay using CBP biotinylated peptides and His-tagged PPARγ LBD (EC_{50} values are shown in **Supplementary Table 4**). (c) Titration of the ligands into protein (as labeled) preloaded with fluorescently labeled ligand (Fluormone Pan-PPAR Green) was used to determine Ki values for ligands to the proteins. Fitted Ki values are displayed in **Supplementary Table 1**. Mean and standard deviation (n=2) are shown along with fitted curve (see methods). Error bars represent the standard deviation between two technical replicates in a single experiment. The competition assay to determine K_i values was performed a single time. The TR-FRET with PPARγ^{K502C}-BTFA and PPARγ^{C313A,K502C}-BTFA was performed three times with similar results. TR-FRET with CBP was performed two times with similar results. (d-f) Comparison of PPARγ, PPARγ^{K502C}-BTFA and PPARγ^{C313A,K502C}-BTFA TR-FRET ratios at saturating ligand concentrations for (d) MED1 (e) CBP and (f) NCoR. Correlation coefficient is indicated (R^2).

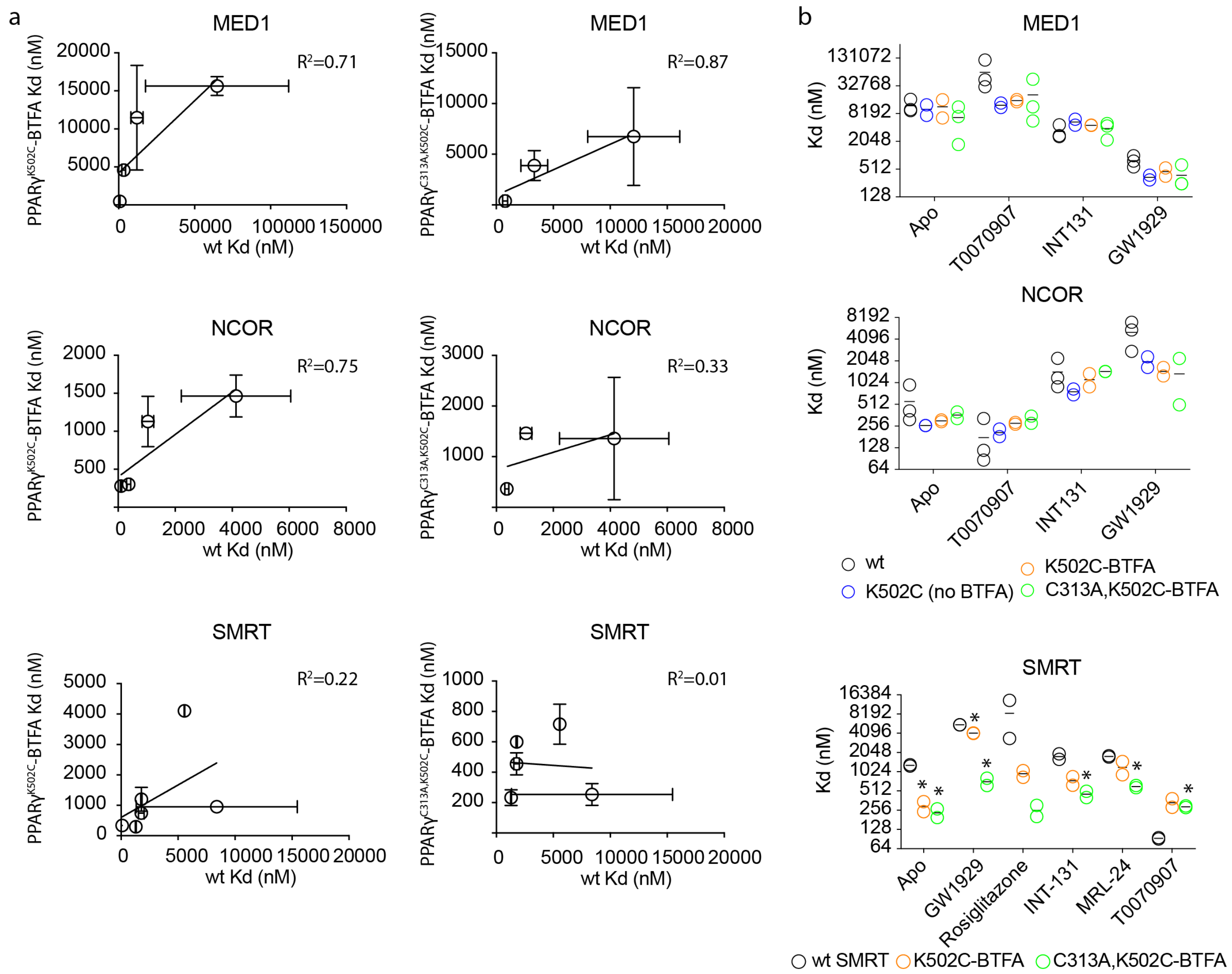

Supplementary Figure 7 | Fluorescence polarization indicates that labeled proteins are functional, that PPARγ^{K502C}-BTFA is most similar to PPARγ and that SMRT binding is perturbed by labeling. (a) Comparison of PPARγ with PPARγ^{K502C}-BTFA and PPARγ^{C313A,K502C}-BTFA affinity for MED1, NCoR and SMRT peptides either without ligand or saturated with T0070907, GW1929 or INT-131 as measured by fluorescence polarization. Assays involving SMRT peptide recruitment also included rosiglitazone and MRL24. Error bars represent standard deviation of 2 (SMRT and wt NCoR) or 3 (PPARγ^{C313A,K502C}-BTFA NCoR and MED1) independently run assays. Correlation coefficient is indicated (R^2). (b) Comparison of peptide affinities for wt and labeled/mutant versions of PPARγ either ligand free or saturated with the indicated ligands. Significant differences between wt and the labeled/mutant PPARγ are indicated by asterisks ($p<0.05$; unpaired t test with Holm-Sidak correction for multiple comparisons).

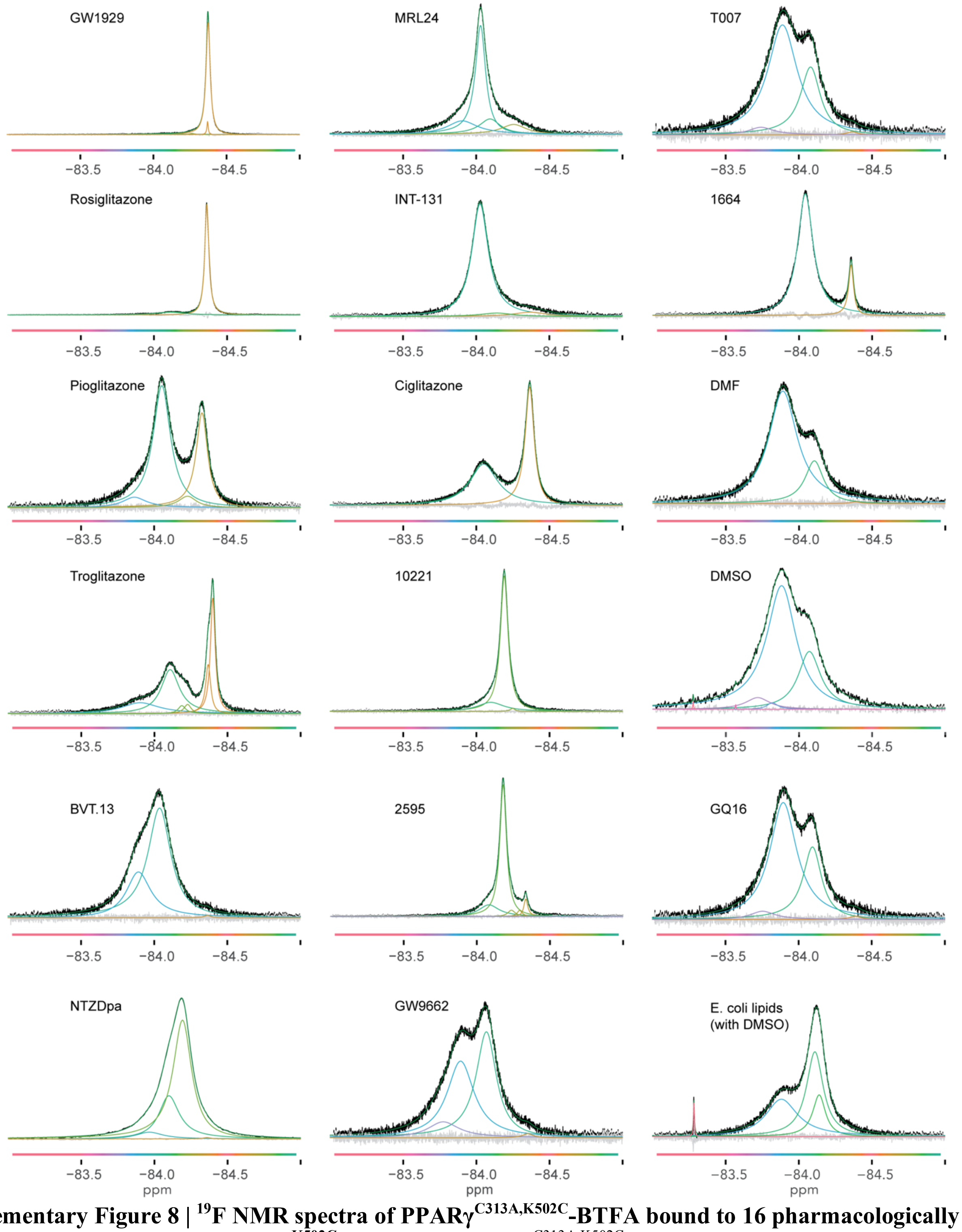

Supplementary Figure 8 | ^{19}F NMR spectra of PPARγ^{C313A,K502C}-BTFA bound to 16 pharmacologically distinct ligands is similar to PPARγ^{K502C}-BTFA. The PPARγ^{C313A,K502C}-BTFA spectra are higher signal to noise than the PPARγ^{K502C}-BTFA spectra because the C313A mutation allows complete labeling with higher BTFA concentrations without spurious labeling of C313 (**Supplementary Fig. 4**). GQ16 used DMF for a vehicle. All other ligands used DMSO. DMSO and DMF concentrations are the same in all spectra. This DMSO spectrum is also displayed in other figures in this manuscript. The estimated degree of saturation with ligand in these spectra is shown in **Supplementary Table 2**. Some of these spectra were replicated (**Supplementary Fig. 10**)

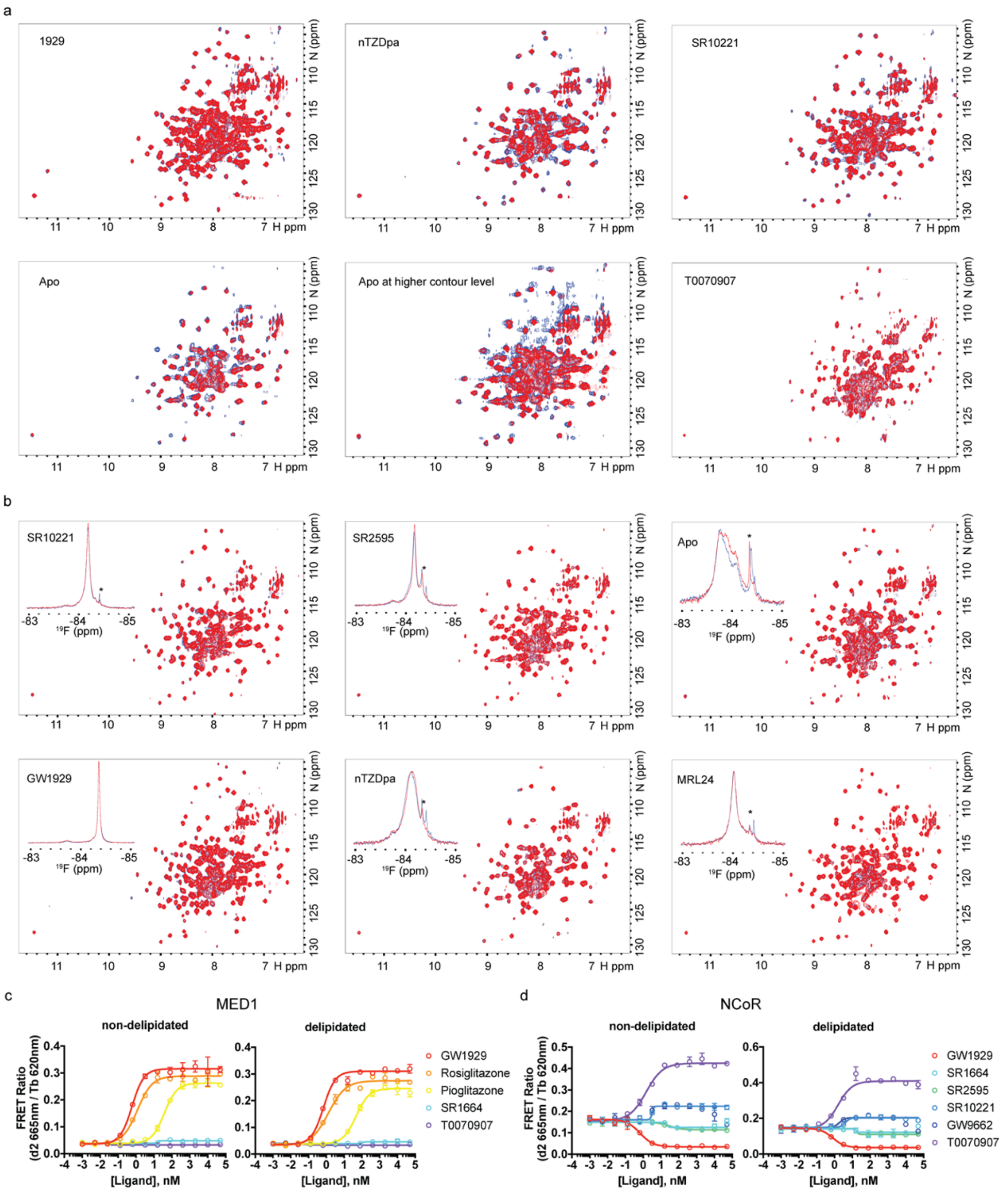

Supplementary Figure 9 | NMR indicates that delipidation produces a small difference in backbone structure and in coregulator recruitment. (a-b) Protein was delipidated (red spectra) or not (blue spectra) and then loaded with the indicated ligand or not loaded with ligand (Apo). 2D [^{1}H,^{15}N]-TROSY-HSQC NMR spectra (Bruker pulse program trosyf3gpphsi19.2) of (a) ^{15}N-labeled PPARγ LBD or (b) ^{15}N-labeled PPARγ^{C313A}-BTFA. Apo spectra are shown at two different contour levels in panel a for clarity of changes vs. liganded states. Insets in panel b show ^{19}F NMR spectra of the same sample. Sharp upfield shifted peaks marked with an asterisk in panel b are likely misfolded protein or another artifact. (c) PPARγ LBD was delipidated or not delipidated prior to measuring coregulator recruitment in response to ligand titration via TR-FRET. No significant difference is observed. Mean ± SE shown. Error bars represent the standard deviation of two technical replicates. TR-FRET was repeated in two independent experiments with similar results. NMR was done once.

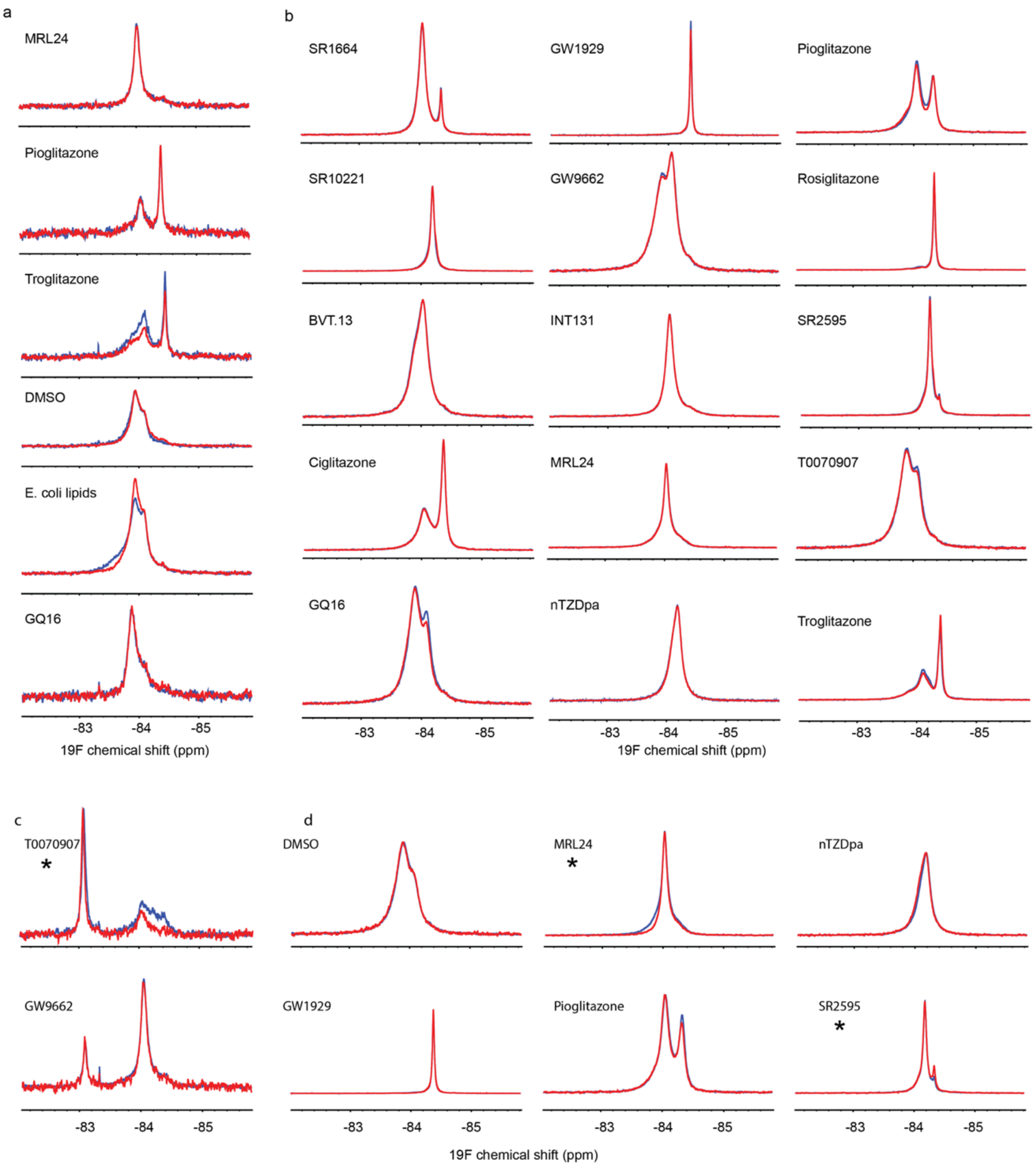

Supplementary Figure 10 | NMR samples are stable over time and replicate samples from different protein preparations produce very similar spectra. Fluorine NMR was collected on (a) PPARγ^{K502C}-BTFA or (b) PPARγ^{C313A,K502C}-BTFA loaded with the indicated ligands initially (blue) and then after two to nine days of storage at room temperature (red). The changes in the *E. coli* lipid panel are likely due to lipid precipitation after unbinding from PPARγ. The blue spectra are displayed in other figures in this manuscript. (c-d) Replicate samples from the same or different protein preparations. (c) replicate samples using PPARγ^{K502C}-BTFA and (d) PPARγ^{C313A,K502C}-BTFA (* denote samples from different protein preparations). Spectra are scaled relative to each other for ease of comparison. All of the spectra in panels c and d are shown elsewhere in this manuscript except for one of the spectra each of pioglitazone and nTZDpa bound to PPARγ^{C313A}-BTFA.

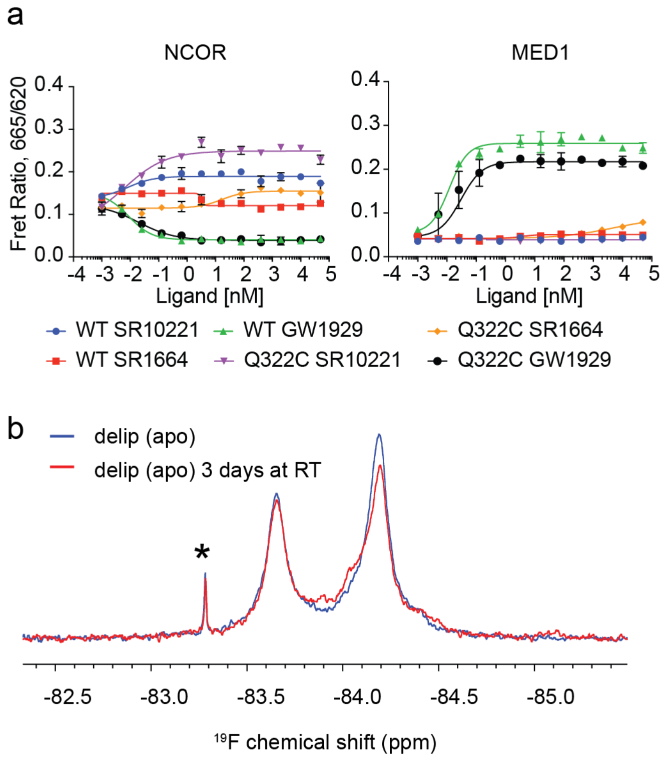

Supplementary Figure 11 | PPARγ^{C313A,Q322C}-BTFA recruits coregulators with similar efficacy to that of wild type PPARγ and the apo PPARγ^{C313A,Q322C}-BTFA spectrum is stable over time. (a) TR-FRET ratio change with increased ligand concentration indicates a change in affinity between the indicated peptide and delipidated PPARγ^{C313A,Q322C}-BTFA induced by ligand binding. (b) ^{19}F NMR spectra of delipidated apo PPARγ^{C313A,Q322C}-BTFA initially and then after 3 days at ambient temperature. The * indicates free BTFA signal. Error bars represent the standard deviation of two technical replicates in a single experiment. TR-FRET was performed twice in two independent experiments with similar results.

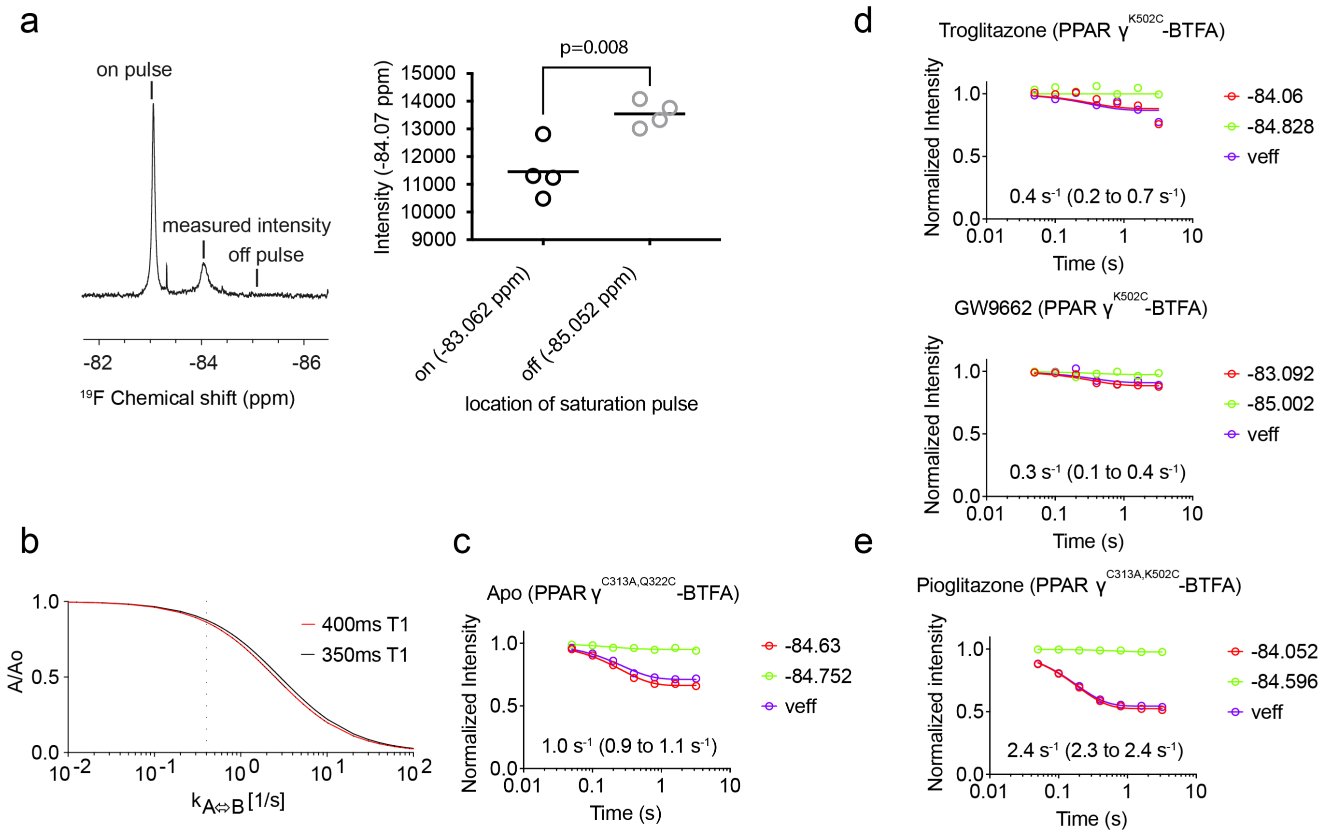

Supplementary Figure 12 | Chemical exchange occurs between ^{19}F NMR resolved peaks. Chemical Exchange Saturation Transfer (CEST) was performed on probes on helix 12 (a,d,e) and on helix 3 (c). (a) Both on (-83.062 ppm) and off (-85.052 ppm) resonance selective saturating pulses were performed and the height of the smaller peak (-84.07 ppm) in the ^{19}F spectrum of T0070907-bound PPARγK502C-BTFA was monitored (left panel). The p value of a two-tailed t-test comparing the mean intensity (shown) of the smaller peak with on and off resonance selective pulses is shown. While exchange is detected, the exchange is likely very slow, resulting in very small changes in intensity of the small peak. (b) Calculation to determine the detection limits of CEST in our system. The intensity of a peak (peak A) as a function of the rate of exchange with a peak that is selectively saturated (peak B) normalized by the intensity of peak A in the absence of exchange (Ao). Calculations are shown using T1 values of 400 ms (red) and 350 ms (black) and a 1.6 second selective saturation pulse (which is what was used in the CEST figures). Calculation was done using Equation 50 from (Journal of Biomolecular NMR 2000 18:49-63). Given experimental noise and peak A and B sharpness in our experiments exchange may be difficult to reliably detect with exchange rates less than ~0.4 s^{-1} (I/Io>0.86; indicated by dotted vertical line in graph). In addition, exchange rates faster than 10 s^{-1} can be detected but may be difficult to quantify given that we used 50 ms selective pulses. (c-e) Exchange rates are indicated with 95% confidence interval in parentheses. An off resonance soft pulse equidistant from the peak of interest (green) was subtracted from an on resonance soft pulse (red) to yield Veff (purple). Fits were carried out fixing T_1 at the experimentally verified rate (**Supplementary Table 5**) and experimentally obtained non-exchange intensity. Thus, only one parameter was fit leaving 6 degrees of freedom in each fit. These experiments were performed once.

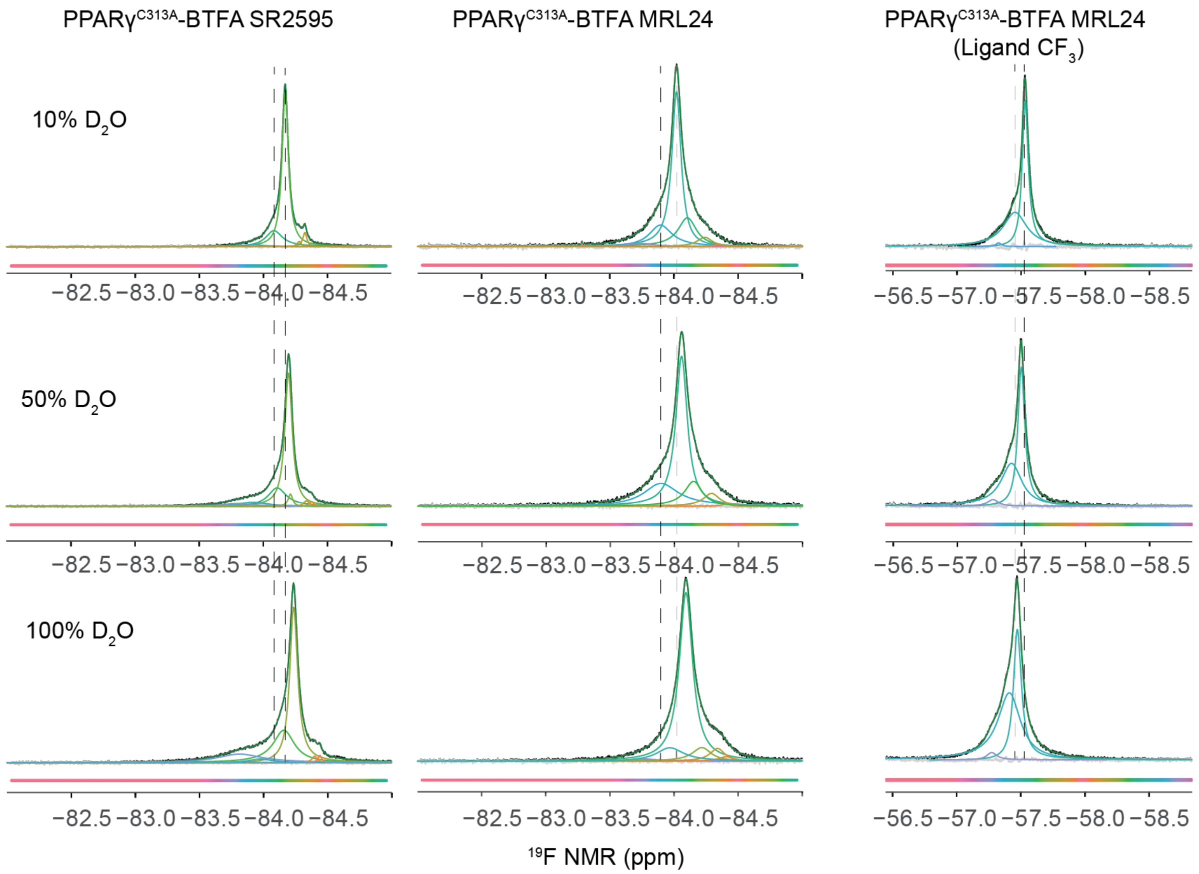

Supplementary Figure 13 | ^{19}F NMR was performed on samples of the indicated protein and ligand complexes in the presence of 10% (upper panel), 50% (middle panel), or 100% (lower panel) deuterium oxide (D_2O). Vertical lines indicate the chemical shift of select peaks at 10% D_2O concentration and are included to aid in comparison of peak position between different D_2O concentrations. These experiments were performed once.

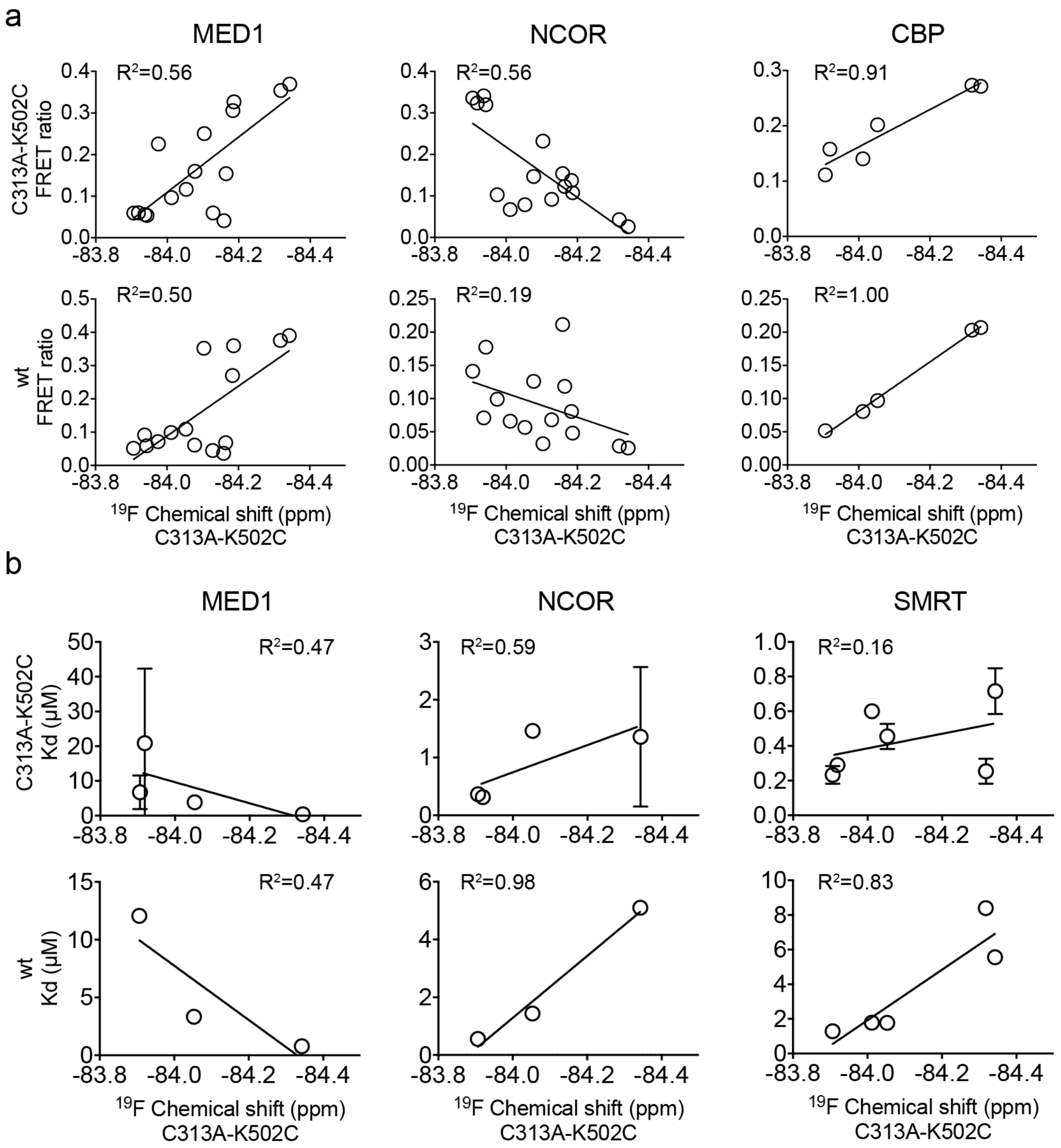

Supplementary Figure 14 | Ligand-directed helix 12 ensemble dictates PPARγ^{C313A,K502C}-BTFA-coregulator interaction. (a) Plot of mean ^{19}F NMR chemical shift values (PPARγ^{C313A,K502C}-BTFA) versus TR-FRET endpoint data for the recruitment of MED1, NCoR and CBP peptides to PPARγ^{C313A,K502C}-BTFA (top panels) and wt PPARγ LBD (bottom panels) for the set of 16 pharmacologically distinct synthetic PPARγ ligands and apo-protein (select ligands for CBP). TR-FRET was performed in three separate experiments for MED1 and NCoR recruitment and two separate experiments for CBP with similar results. b) Plot of mean ^{19}F NMR chemical shift values (PPARγ^{C313A,K502C}-BTFA) versus MED1, NCoR and SMRT peptide dissociation constant (Kd) for PPARγ^{K502C}-BTFA (top panels) and wt PPARγ LBD (bottom panels) as measured by fluorescence polarization (FP) for a subset of the ligands in panel a. Mean and standard deviation for FP represent 2 (NCOR binding to PPARγ^{C313A,K502C}-BTFA and SMRT) or 3 (NCOR binding to wt and MED1) independent replications. Linear regression fit is shown as a solid line and the correlation coefficient (R^2) for the fitted line is indicated. Covalent ligands, which require C313 for binding are not included in the wt graphs as the NMR chemical shift would not be expected to correlate with the FP or TR-FRET values.

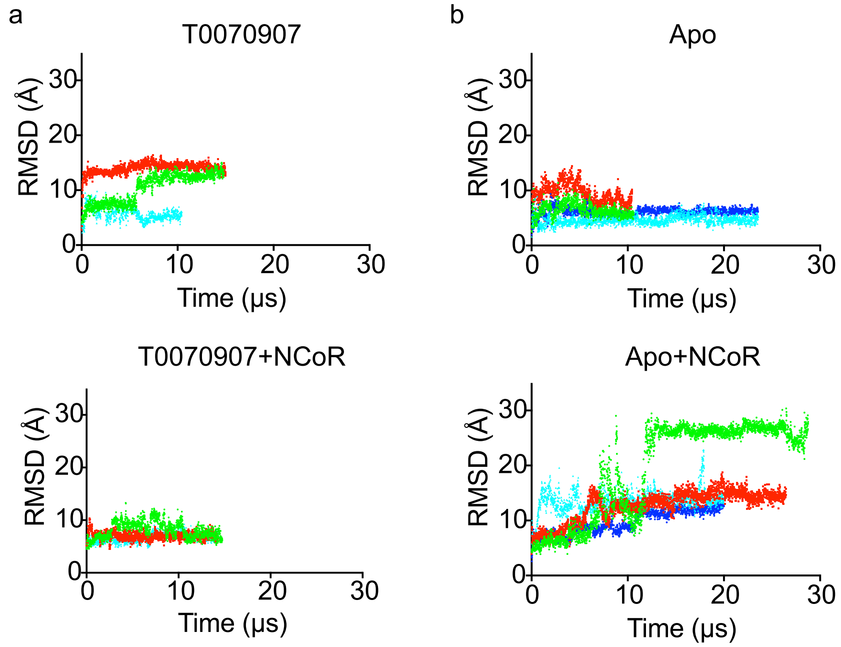

Supplementary Figure 15 | Molecular simulations of PPARγ LBD bound to (a) T0070907 (covalent inverse agonist) alone or cobound to the corepressor peptide NCoR (lower panel) or (b) apo or apo bound to NCoR (lower panel). The root mean square deviation of helix 12 compared to the conformation of helix 12 in the crystal structure that was used to build these molecules (chain B of 3B0R and 1PRG). Each color represents an independent simulation started from the same coordinates but with different initial atom velocities. Apo without NCoR stays the closest to the starting crystal structure.

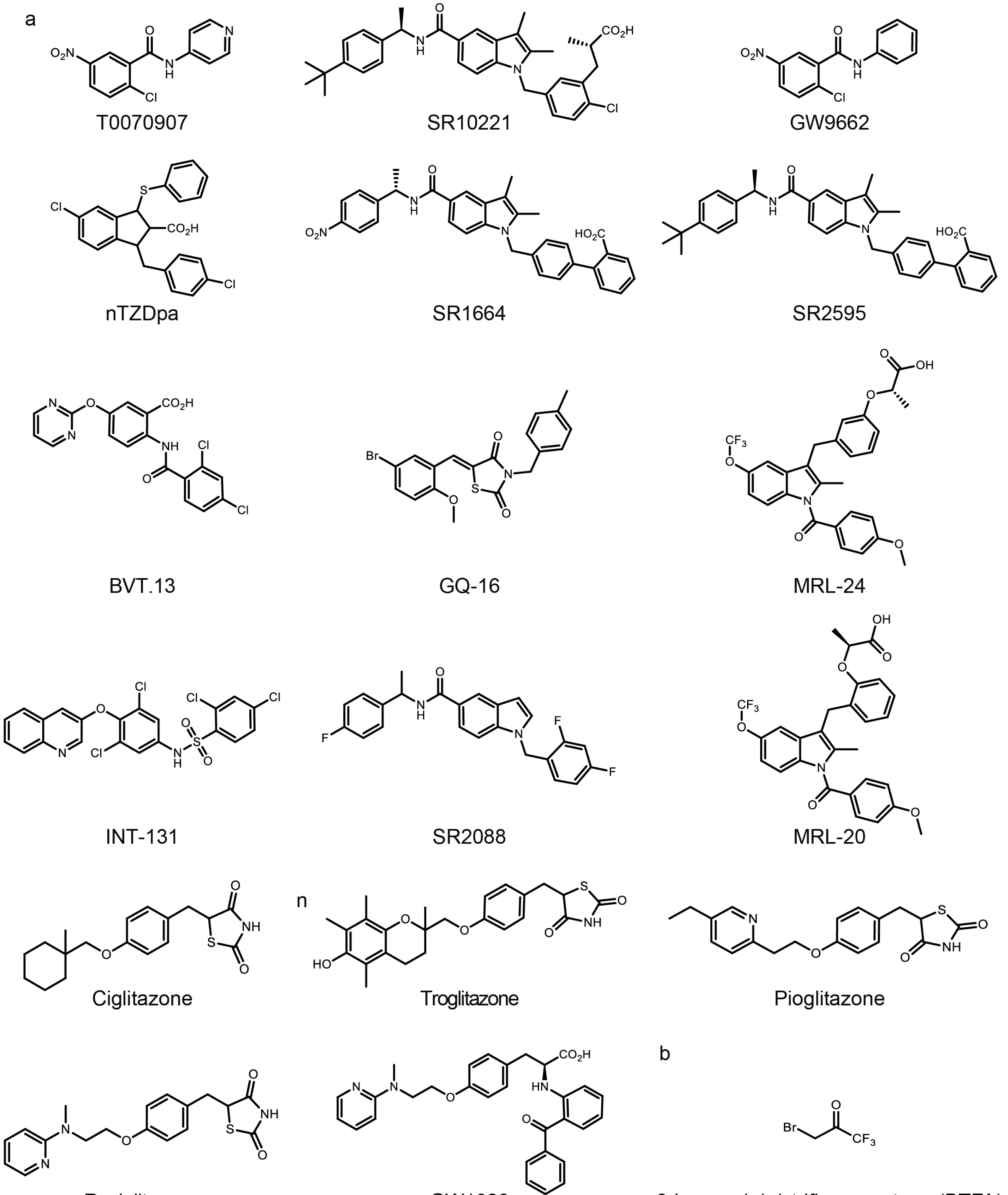

Supplementary Figure 16 | Synthetic PPARγ ligands and ^{19}F label used in this study a) Chemical structures of the ligands used in this study. b) Chemical structure of the 3-bromo-1,1,1-trifluoroacetone label used in this study.

Supplementary Table 1 | Effect of labeling on ligand affinity

Ligand	#EC_{50} (nM)			*K_i (nM)			Fold increase in K_i compared to wild-type	
	wt	PPARγ^{K502C}-BTFA	PPARγ^{C313A,K502C}-BTFA	wt	PPARγ^{K502C}-BTFA	PPARγ^{C313A,K502C}-BTFA	PPARγ^{K502C}-BTFA	PPARγ^{C313A,K502C}-BTFA
Pioglitazone	247	428	412	121	696	1137	6	9
Rosiglitazone	32	81	516	16	132	1422	8	92
Ciglitazone	9930	>50uM	9034	4875	>50uM	24921	36	5
Troglitazone	4699	8723	6387	2306	14177	17619	6	8
INT-131	5	66	22	3	107	60	41	23
nTZDpa	21	19	44	10	31	123	3	12
GQ-16	8481	2171	8873	4163	3528	24477	1	6
BVT.13	10105	7087	7641	4960	11518	21079	2	4
SR10221	292	282	314	143	458	866	3	6
GW1929	2	3	5	1	5	13	5	12
SR2088	33	45	68	16	74	189	5	12
SR2595	110	80	352	54	130	972	2	18
^GW9662	6	14	33204	NA	NA	NA	similar EC_{50}	large change in EC_{50}
^T0070907	15	25	>50uM	NA	NA	NA	similar EC_{50}	large change in EC_{50}
SR1664	131	74	551	64	120	1519	2	24
MRL24	0.9	1.3	1.4	0.5	2	4	5	8
						median	5	11
						mean	9	17

NA = Not applicable

#Given that 8nM protein was used in determining EC_{50} values in this table any EC_{50} less than 4 nM indicates that the true EC_{50} is ≤ 4 nM.

^these are covalent ligands therefore EC50 doesn't have the same meaning as the non-covalent ligands

Supplementary Table 2 | *Fraction of protein bound by ligand for NMR samples*

	Assumes all added ligand is soluble.		Assumes 10 uM solubility of ligand.		Fraction bound according to deconvolution of NMR signal	
	PPARγ-BTFA	PPARγ^{C313A}-BTFA	PPARγ-BTFA	PPARγ^{C313A}-BTFA	PPARγ-BTFA	PPARγ^{C313A}-BTFA
Pioglitazone	1.00	0.99	0.96	0.94	1.00	0.89
Rosiglitazone	1.00	0.94	0.99	0.93	1.00	0.95
Ciglitazone	0.55	0.81	0.37	0.69	*1.00	*0.96
Troglitazone	0.92	0.85	0.76	0.74	0.74	0.81
INT-131	1.00	1.00	0.99	0.99	1.00	1.00
nTZDpa	1.00	0.99	1.00	0.99	1.00	1.00
GQ-16	0.93	0.71	0.89	0.69	#0.57	#0.29
BVT.13 Pentahydrate	0.84	0.73	0.79	0.71	0.65	0.69
SR10221	0.99	0.96	0.97	0.95	1.00	1.00
GW1929	1.00	1.00	1.00	1.00	1.00	1.00
SR2088	1.00	0.99	0.99	0.98	1.00	ND
SR2595	1.00	0.96	0.99	0.95	1.00	1.00
^GW9662	1.00	NA	1.00	NA	0.90	NA
^T0070907	1.00	NA	1.00	NA	1.00	NA
SR1664	1.00	0.94	0.99	0.93	1.00	1.00
MRL24	1.00	1.00	1.00	1.00	1.00	0.86
Vehicle (DMSO)					##0.21	##0.25

Fraction bound is calculated using Ki values for ligands.
**Discrepancy between NMR and calcultation is likely a combination of inaccurate deconvolution and good solubility of ciglitazone (Cayman Chemical states that ciglitazone is soluble to 1.2mM in 25% DMSO).*
^Covalent ligands were added at 2x and so are assumed to be fully bound based on EC50 values, this is supported by LC-ESI MS data.
# GQ16 has very poor solubility, which would explain the low bound fraction observed via NMR.
## This is either residual bound E. coli lipid or an Apo conformation that is shifted to the right.

Supplementary Table 3 | EC_{50} values for MED1 and NCOR recruitment

Coregulator	MED1 EC_{50} (nM)**					NCOR EC_{50} (nM)**				
PPARγ variant	wt	$PPAR\gamma^{K502C}$-BTFA	FC	$PPAR\gamma^{C313A,K502C}$-BTFA	FC	wt	$PPAR\gamma^{K502C}$-BTFA	FC	$PPAR\gamma^{C313A,K502C}$-BTFA	FC
Pioglitazone	190	420	2	970	5	350	2200	6	1,200	3
Rosiglitazone	13	29	2	99	8	21	96	5	1,200	57
Ciglitazone	4200	7400	2	1800	<1	2,700	15,000	6	14,000	5
Troglitazone	580	2600	4	1400	2	1,100	6,600	6	4,600	4
INT-131	7	1	<1	18	2	2	3	1	38	19
*nTZDpa**	8	<1	<1	<1	<1	4	8	2	47	12
GQ-16	730	1600	2	NC	NA	1,900	1,900	1	NC	NA
BVT.13 Pentahydrate	>50,000	>50,000	NA	14,000	<1	44,000	9,300	<1	19,000	<1
SR10221	8	NC	NA	4	<1	2	28	14	28	14
GW1929	3	4	1	3	1	2	10	5	91	46
SR2088	23	50	2	37	1	13	34	3	120	9
SR2595	100	NC	NA	680	7	75	34	<1	590	8
GW9662#	4	NC	NA	NC	NA	3	37	12	NC	NA
T0070907#	4	NC	NA	>50,000	NA	13	66	5	NC	NA
SR1664	180	NC	NA	5600	31	NC	99	NA	535	NA
*MRL24**	2	1	<1	1	<1	<1	1	1	2	2
Median change§			2		1			5		9
Mean change§			2		5			5		15

NA=not applicable
NC=no significant change in TR-FRET ratio was observed, which does not allow curve fitting, which is expected for non-agonists and for non-agonists and inverse agonists in the MED1 assay.
*Data indicates two recruitment events. The EC50 is shown for the initial peptide recruitment event only. The second recruitment event likely is caused by binding of a second ligand to the same PPARγ molecule[1].
these ligands form a covalent adduct to C313 and should not bind to $PPAR\gamma^{C313A,K502C}$-BTFA.
**Given that 8nM protein was used in determining EC_{50} values in this table any EC_{50} less than 4 nM indicates that the true EC_{50} is ≤ 4 nM.
§not including covalent ligands; values less than 1 (<1) are treated as 1

Supplementary Table 4 | EC_{50} values for CBP recruitment

Coregulator	CBP EC_{50} (nM)**				
PPARγ variant	wt	PPARγ^{K502C}-BTFA	Fold change	PPARγ^{C313A,K502C}-BTFA	Fold change
Rosiglitazone	2	4	2	11	8
INT-131	<1	<1	1	<1	1
GW1929	<1	<1	1	<1	1
T0070907#	2	4	2	41,000	21,000
*MRL24**	<1	1	1	1	1
Median change			1		§1
Mean change			1.4		§2.8

*Data indicates two recruitment events. The EC50 is shown for the initial peptide recruitment event only. The second recruitment event likely is caused by binding of a second ligand to the same PPARγ molecule[1].
this ligand forms a covalent adduct to C313 and should not bind to PPARγ^{C313A,K502C}-BTFA.
**Given that 8nM protein was used in determining EC_{50} values in this table any EC_{50} less than 4 nM indicates that the true EC_{50} is ≤ 4 nM.
§not including covalent ligands; values less than 1 (<1) are treated as 1

Supplementary Table 5 | NMR derived spin relaxation values are consistent with deconvolution

Ligand, protein and peak	T_2 value (mean and 95% confidence interval: ms)	Predicted FWHM (Hz) from T_2 value 95% CI ($1/\pi * T_2$)	Deconvolution FWHM (Hz)*	T_1 value (mean and 95% confidence interval: ms)
Pioglitazone *PPARγ^{C313A,K502C}-BTFA* *-84.06 ppm*	4.9 (4.6 to 5.3)	47 to 54	95	365 (348 to 382)
Pioglitazone *PPARγ^{C313,K502CA}-BTFA* *-84.33 ppm*	6.2 (5.7 to 6.7)	38 to 46	62	367 (356 to 379)
GW1929 *PPARγ^{C313A,K502C}-BTFA* *-84.38 ppm*	17.6 (16.9 to 18.4)	16 to 18	22	342 (339 to 344)
Apo *PPARγ^{C313A,K502C}-BTFA* *-84.06 ppm*	3.1 (2.9 to 3.4)	94 to 110	112	404 (360 to 451)
Apo *PPARγ^{C313A,K502C}-BTFA* *-83.9 ppm*	2.2 (2.0 to 2.3)	135 to 152	174	415 (396 to 434)

**Decovoluted peak widths are all slightly wider than the T_2 predicted linewidth — if they were narrower, this would indicate over-fitting of the data. Thus, the deconvolution provides a fit with the least number of possible peaks (i.e. the number of chemical environments, or conformations) sampled by the BTFA probe attached to helix 12 for these spectra.*

Supplementary Table 6 | Primer sequences* used for mutagenesis in this study

K502C	5’-aggttaattattagtacaagtcgcagtagatctcctgcaggagcgg-3’
C313A	5’-ccacggagcgaaactgagcgccctgaaagatgcgg-3’
Q322C	5’-gcatactctgtgatctcgcacacagcctccacggagc-3’
Y505C	5’-gagatctacaaggacttgtgctaacccgggcttctcctc-3’
C313S	5’-atccgcatctttcagggctctcagtttcgctccgtggag-3’
Q498C	5’- acaagtccttgtagatctcgcacaggagcgggtgaagactc-3’

*reverse primers are the reverse complement of the sequences shown

Supplementary Note 1 | Parameters used in simulations

```
&cntrl
  imin = 0, nstlim = 250000000, dt=0.004,
  ntx = 5, irest = 1,
  ntwx = 25000, ioutfm = 1, ntxo =2, ntpr = 25000, ntwr = 25000,
  iwrap = 1, nscm = 1000,
  ntc = 2, ntf = 2, ntb = 2, cut = 8.0,
  ntt = 3, ig = -1, gamma_ln = 3, temp0 = 310, tempi = 310,
  ntp = 1, taup = 2.0, barostat = 2,
  igb = 0, saltcon = 0.0,
  ntr = 0,
```

Supplementary Note 2 | Prepin file

```
    0    0    2

This is a remark line
molecule.res
CYB   INT  0
CORRECT     OMIT DU   BEG
  0.0000
   1  DUMM  DU    M    0  -1  -2     0.000      .0        .0      .00000
   2  DUMM  DU    M    1   0  -1     1.449      .0        .0      .00000
   3  DUMM  DU    M    2   1   0     1.523   111.21       .0      .00000
   4  N     N     M    3   2   1     1.540   111.208  -180.000 -0.415700
   5  H     H     E    4   3   2     1.001   128.253    75.116  0.271900
   6  CA    CT    M    4   3   2     1.453    46.684    -6.789  0.021300
   7  HA    H1    E    6   4   3     1.085   106.975   -51.989  0.112400
   8  CB    CT    3    6   4   3     1.532   114.777    70.403 -0.052400
   9  HB2   H1    E    8   6   4     1.083   111.004   -48.445  0.125000
  10  HB3   H1    E    8   6   4     1.080   109.153    70.321  0.125000
  11  SG    S     S    8   6   4     1.820   113.917  -171.908 -0.275000
  12  CD    CT    3   11   8   6     1.812    99.158    84.434 -0.125700
  13  HD2   H1    E   12  11   8     1.085   111.557   -58.090  0.067200
  14  HD3   H1    E   12  11   8     1.085   110.619    60.963  0.067200
  15  CE    C     B   12  11   8     1.510   111.238  -178.762  0.509200
  16  O1    O     E   15  12  11     1.181   125.861    -0.134 -0.397100
  17  CZ    CT    3   15  12  11     1.537   114.766   179.713  0.511800
  18  F3    F     E   17  15  12     1.323   109.977    58.455 -0.191500
  19  F1    F     E   17  15  12     1.305   111.986   179.382 -0.191500
  20  F2    F     E   17  15  12     1.324   109.860   -59.791 -0.191500
  21  C     C     M    6   4   3     1.534   104.761  -167.409  0.597300
  22  O     O     E   21   6   4     1.192   120.251   -90.053 -0.567900


LOOP
```

```
IMPROPER
  CD   CZ   CE   O1
  CA   +M    C    O

DONE
STOP
```

Supplementary Note 3 | AMBER parameter database derived frcmod file

```
Remark line goes here
MASS
N  14.010       0.530
H  1.008        0.161
CT 12.010       0.878
H1 1.008        0.135
S  32.060       2.900
C  12.010       0.616
O  16.000       0.434
F  19.000       0.320

BOND
H -N   434.00   1.010
CT-N   337.00   1.449
CT-H1  340.00   1.090
CT-CT  310.00   1.526
C -CT  317.00   1.522
CT-S   227.00   1.810
C -O   570.00   1.229
CT-F   367.00   1.380

ANGLE
H1-CT-N    50.000     109.500
CT-CT-N    80.000     109.700
C -CT-N    63.000     110.100
CT-N -H    50.000     118.040
CT-CT-H1   50.000     109.500
CT-CT-S    50.000     114.700
CT-C -O    80.000     120.400
C -CT-H1   50.000     109.500
C -CT-CT   63.000     111.100
CT-S -CT   62.000      98.900
H1-CT-H1   35.000     109.500
H1-CT-S    50.000     109.500
CT-C -CT   63.000     117.000
F -CT-F    77.000     109.100
```

DIHE
H1-CT-CT-N   9   1.400   0.000   3.000
N -CT-CT-S   9   1.400   0.000   3.000
O -C -CT-N   6   0.000   0.000   2.000
H1-CT-N -H   6   0.000   0.000   2.000
CT-CT-N -H   6   0.000   0.000   2.000
C -CT-N -H   6   0.000   0.000   2.000
CT-CT-S -CT   3   1.000   0.000   3.000
H1-CT-CT-H1   9   1.400   0.000   3.000
H1-CT-CT-S   9   1.400   0.000   3.000
O -C -CT-H1   1   0.800   0.000   -1.000
O -C -CT-H1   1   0.000   0.000   -2.000
O -C -CT-H1   1   0.080   180.000   3.000
O -C -CT-CT   6   0.000   0.000   2.000
H1-CT-S -CT   3   1.000   0.000   3.000
C -CT-S -CT   3   1.000   0.000   3.000
C -CT-CT-H1   9   1.400   0.000   3.000
C -CT-CT-S   9   1.400   0.000   3.000
O -C -CT-S   6   0.000   0.000   2.000
CT-C -CT-S   6   0.000   0.000   2.000
CT-C -CT-F   6   0.000   0.000   2.000
CT-C -CT-H1   6   0.000   0.000   2.000
O -C -CT-F   6   0.000   0.000   2.000

IMPROPER
CT-CT-C -O   10.5   180.0   2.0   Using general improper torsional angle X- X- C- O, penalty score= 6.0)

NONBON
N   1.8240 0.1700
H   0.6000 0.0157
CT   1.9080 0.1094
H1   1.3870 0.0157
S   2.0000 0.2500
C   1.9080 0.0860
O   1.6612 0.2100
F   1.7500 0.0610

Supplementary Note 4 | GAFF parameter database derived frcmod file

Remark line goes here
MASS
N 14.010   0.530   same as n
H 1.008   0.161   same as hn
CT 12.010   0.878   same as c3
H1 1.008   0.135   same as h1
S 32.060   2.900   same as ss
C 12.010   0.616   same as c

O 16.000 0.434 same as o
F 19.000 0.320 same as f

BOND
H -N   403.20   1.013   same as hn- n, penalty score= 0.0
CT-N   328.70   1.462   same as c3- n, penalty score= 0.0
CT-H1 330.60   1.097   same as c3-h1, penalty score= 0.0
CT-CT 300.90   1.538   same as c3-c3, penalty score= 0.0
C -CT 313.00   1.524   same as c-c3, penalty score= 0.0
CT-S   215.90   1.839   same as c3-ss, penalty score= 0.0
C -O   637.70   1.218   same as c- o, penalty score= 0.0
CT-F   356.90   1.350   same as c3- f, penalty score= 0.0

ANGLE
H1-CT-N   49.840   108.880   same as h1-c3-n , penalty score= 0.0
CT-CT-N   65.910   111.610   same as c3-c3-n , penalty score= 0.0
C -CT-N   67.000   109.060   same as c -c3-n , penalty score= 0.0
CT-N -H   45.800   117.680   same as c3-n -hn, penalty score= 0.0
CT-CT-H1   46.390   109.560   same as c3-c3-h1, penalty score= 0.0
CT-CT-S   61.300   110.270   same as c3-c3-ss, penalty score= 0.0
CT-C -O   67.400   123.200   same as c3-c -o , penalty score= 0.0
C -CT-H1   47.040   108.220   same as c -c3-h1, penalty score= 0.0
C -CT-CT   63.270   111.040   same as c -c3-c3, penalty score= 0.0
CT-S -CT   60.240   99.240   same as c3-ss-c3, penalty score= 0.0
H1-CT-H1   39.240   108.460   same as h1-c3-h1, penalty score= 0.0
H1-CT-S   42.060   108.760   same as h1-c3-ss, penalty score= 0.0
C -CT-S   61.850   108.840   same as c -c3-ss, penalty score= 0.0
CT-C -CT   62.040   116.500   same as c3-c -c3, penalty score= 0.0
C -CT-F   66.260   110.000   same as c -c3-f , penalty score= 0.0
F -CT-F   70.890   107.360   same as f -c3-f , penalty score= 0.0

DIHE
H1-CT-CT-N   9   1.400   0.000   3.000   same as X -c3-c3-X , penalty score= 0.0
N -CT-CT-S   9   1.400   0.000   3.000   same as X -c3-c3-X , penalty score= 0.0
O -C -CT-N   6   0.000   180.000   2.000   same as X -c -c3-X , penalty score= 0.0
H1-CT-N -H   6   0.000   0.000   2.000   same as X -c3-n -X , penalty score= 0.0
CT-CT-N -H   6   0.000   0.000   2.000   same as X -c3-n -X , penalty score= 0.0
C -CT-N -H   6   0.000   0.000   2.000   same as X -c3-n -X , penalty score= 0.0
CT-CT-S -CT   3   1.000   0.000   3.000   same as X -c3-ss-X , penalty score= 0.0
H1-CT-CT-H1   9   1.400   0.000   3.000   same as X -c3-c3-X , penalty score= 0.0
H1-CT-CT-S   9   1.400   0.000   3.000   same as X -c3-c3-X , penalty score= 0.0
O -C -CT-H1   1   0.800   0.000   -1.000   same as h1-c3-c -o
O -C -CT-H1   1   0.000   0.000   -2.000   same as h1-c3-c -o
O -C -CT-H1   1   0.080   180.000   3.000   same as h1-c3-c -o , penalty score= 0.0
O -C -CT-CT   6   0.000   180.000   2.000   same as X -c -c3-X , penalty score= 0.0
H1-CT-S -CT   3   1.000   0.000   3.000   same as X -c3-ss-X , penalty score= 0.0
C -CT-S -CT   3   1.000   0.000   3.000   same as X -c3-ss-X , penalty score= 0.0

C -CT-CT-H1	9	1.400	0.000	3.000	same as X -c3-c3-X , penalty score= 0.0
C -CT-CT-S	9	1.400	0.000	3.000	same as X -c3-c3-X , penalty score= 0.0
O -C -CT-S	6	0.000	180.000	2.000	same as X -c -c3-X , penalty score= 0.0
CT-C -CT-S	6	0.000	180.000	2.000	same as X -c -c3-X , penalty score= 0.0
CT-C -CT-F	6	0.000	180.000	2.000	same as X -c -c3-X , penalty score= 0.0
CT-C -CT-H1	6	0.000	180.000	2.000	same as X -c -c3-X , penalty score= 0.0
O -C -CT-F	6	0.000	180.000	2.000	same as X -c -c3-X , penalty score= 0.0

IMPROPER

CT-CT-C -O	1.1	180.0	2.0	Using the default value

NONBON

N	1.8240	0.1700	same as n
H	0.6000	0.0157	same as hn
CT	1.9080	0.1094	same as c3
H1	1.3870	0.0157	same as h1
S	2.0000	0.2500	same as ss
C	1.9080	0.0860	same as c
O	1.6612	0.2100	same as o
F	1.7500	0.0610	same as f

Supplementary References

1. Hughes, T. S. *et al.* An alternate binding site for PPARγ ligands. *Nat. Commun.* **5,** 3571 (2014).
